# Investing in future pediatric subspecialists: a fellowship curriculum that prepares for the transition to academic careers

**DOI:** 10.3402/meo.v20.26714

**Published:** 2015-04-08

**Authors:** Jennifer A. Rama, Judith R. Campbell, Dorene F. Balmer, Teri L. Turner, Deborah C. Hsu

**Affiliations:** Department of Pediatrics, Baylor College of Medicine, Texas Children's Hospital, Houston, TX, USA

**Keywords:** academic career development, pediatric subspecialties, fellowship training, career guidance, non-clinical skills

## Abstract

**Background:**

The experience of transitioning to an academic faculty position can be improved with standardized educational interventions. Although a number of such interventions have been described, few utilize an evaluation framework, describe a robust evaluation process, and address why their interventions were successful. In this article, the authors apply a logic model to describe their efforts to develop, implement, evaluate, and revise a comprehensive academic career development curriculum among pediatric subspecialty fellows. They describe inputs, activities, outputs, and outcomes using quantitative data from fellow evaluations and qualitative data from faculty interviews.

**Methods:**

Methods are described under the input and activities sections. The curriculum started with collaboration among educational leadership and conducting a needs assessment. Using the needs assessment results and targeted learning objectives, we piloted the curriculum and then implemented the full curriculum 1 year later.

**Results:**

Results are described under the outputs and outcomes sections. We present immediate, short-term, and 6-month evaluation data. Cumulative data over 3 years reveal that fellows consistently acquired knowledge relevant to transitioning and that they applied acquired knowledge to prepare for finding jobs and career advancement. The curriculum also benefits faculty instructors who gain a sense of reward by filling a critical knowledge gap and fostering fellows’ professional growth.

**Conclusion:**

The authors relate the success and effectiveness of the curriculum to principles of adult learning, and share lessons learned, including the importance of buy-in from junior and senior fellows and faculty, collaboration, and designating the time to teach and learn.

The transition from subspecialty fellow to attending physician may be complicated by lack of competence in critical non-clinical skills. Existing articles describe the need to improve management, teaching, and leadership skills (1) and that a lack of academic preparedness leads to a more stressful and challenging transition (2, 3). The need to prepare for academia early in training is increasingly recognized, and various educational interventions such as workshops, retreats, and formal programs have been described (4–10). These articles suggest that fellows benefit from standardized educational interventions designed to prepare them for academic careers. However, few articles describe a robust evaluation process to study not only *did it work?* but *why did it work?*

As curriculum directors and educational leaders in the Department of Pediatrics at Baylor College of Medicine (BCM), we recognized that most of our fellows pursue academic positions. Historically, fellows received informal instruction on how to prepare for academic careers and the quality and type of guidance varied widely across divisions within the department. Therefore, we created the BCM, Department of Pediatrics, Academic Career Development (ACD) Curriculum which was offered to all clinical pediatric subspecialty fellows. The goal of the curriculum is to facilitate smooth transitions to academic faculty positions for graduates who desire this career path.

In this article, we describe our efforts to develop, implement, evaluate, and revise the BCM, Department of Pediatrics, ACD Curriculum. We used a logic model to frame our efforts, as it provides a roadmap for curricula as a whole and depicts how component parts logically flow toward the desired learning outcome (11, 12) (Fig. 1). Absent from most existing articles on career development, we describe evaluation data from multiple perspectives using both quantitative and qualitative data and also relate principles of adult learning to explain why the curriculum has been a success.

**Fig. 1:**
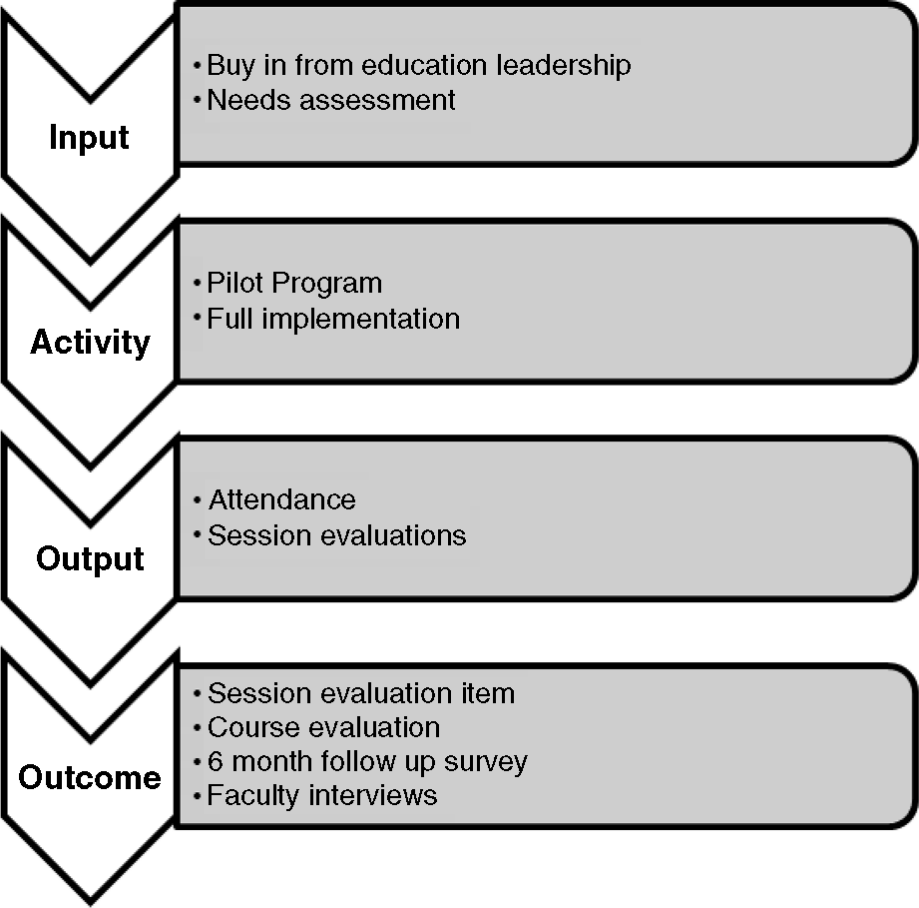
Logic model.

## Inputs

Inputs are the resources we utilized in developing and implementing the curriculum.

### Educational leadership

Education leaders in the BCM Department of Pediatrics, including the director of fellowship education, the executive vice chair, vice chair, and associate vice chairs for education, supported early proposals for a departmental ACD curriculum. To that end, they contributed time and expertise in planning the curriculum and included it as part of Fellows’ College (13), an established centralized program in the Department that offers non-specialty specific curricula. Through Fellows College, we had access to administrative support and funding to cover the cost of lunches.

### Needs assessment

We conducted a needs assessment to determine buy-in from two key stakeholder groups: fellowship program directors (PDs) and associate program directors (APDs), and pediatric subspecialty fellows. We asked about the 1) usefulness of 12 topics related to transition in academic medicine, 2) perceived knowledge of these topics, 3) whether current instruction in career development needed improvement 4) perceived benefit of a formalized curriculum.

We administered the needs assessment survey to all PDs and APDs (*n*=33) and all clinical pediatric subspecialty fellows (*n*=150). Of these key stakeholder groups, 82% of PDs and APDs responded, representing 21 pediatric subspecialties. Thirty-eight fellows responded, representing 15 subspecialties and all years of training (42, 32, and 26% were first-, second-, and third-year fellows, respectively).

As displayed in Table 1, there was an inverse relationship between usefulness and level of knowledge of the 12 topics. That is, topics ranked as most useful to know for transitioning were the same topics about which fellows were least knowledgeable. Almost 75% of respondents indicated that career guidance in their division either needed improvement or was inadequate. Similarly, the majority of respondents indicated that a formalized curriculum in career guidance would be beneficial.

**Table 1:** Faculty assessment of the level of usefulness of 12 topics and of fellows’ level of knowledge in those topics

	Faculty responses			Fellow responses		
	
Topics	Usefulness	Fellows' knowledge	p*	Usefulness	knowledge	p*
1. Medical Employment Contracts	4.21	1.66	<0.001	4.45	2.00	<0.001
2. Process for academic promotion	4.47	2.10	<0.001	4.34	2.42	<0.001
3. Assessment of productivity	4.39	2.17	<0.001	4.39	2.37	<0.001
4. Interviewing/negotiating skills	4.44	2.20	<0.001	4.39	2.55	<0.001
5. Effective writing of grants/publications	4.58	2.38	<0.001	4.37	2.00	<0.001
6. Understanding different academic pathways	4.58	2.66	<0.001	4.29	3.37	<0.001
7. Preparing a curriculum vitae	4.2	2.98	<0.001	4.29	3.37	<0.001
8. Effective goal setting	4.38	2.78	<0.001	4.16	3.21	<0.001
9. Teaching effectively	4.14	3.12	<0.001	4.32	3.24	<0.001
10. Personal organization/time	4.18	2.92	<0.001	3.84	3.37	0.077
11. Managing stress	3.09	2.75	<0.001	3.61	3.42	0.493
12. Mentoring skills	4.18	2.41	<0.001	4.13	2.79	<0.001
All 12 topics	51.64	30.13	<0.001	50.82	33.68	<0.001

Corresponding fellows’ assessment of the level of usefulness and their perceived knowledge in same topics.

Likert scale 0=not useful/not knowledgeable to 5=most useful/most knowledgeable.

* *p*-Value corresponds to comparison between usefulness and level of knowledge.

## Activity

The activity phase involved implementing a pilot course and then the full curriculum. Our study protocol was submitted to the Institutional Review Board and deemed exempt from review.

### Pilot course

We met with a sub-group of PDs to design a pilot course and make decisions about curriculum content, instructional methods, and scheduling which were informed by data from the needs assessment survey. We invited faculty members with expertise to give the following lectures: Pitfalls of Manuscript Writing, Understanding Different Academic Pathways, Basics of Productivity and Promotion, and Medical Employment Contracts. Learning objectives were derived from perceived needs of fellows and from gaps identified by the faculty-experts. In scheduling the pilot curriculum, we considered the availability of conference rooms, faculty schedules, and time of day considered least intrusive of other responsibilities, namely the lunch hour. We conducted the pilot course with subspecialty fellows in four divisions.

### Full curriculum

The following year, we implemented the full, six-session course for all pediatric subspecialty fellows. Two additional sessions, namely Successful Grant Writing and Interviewing/Negotiating were added to pilot sessions. We invited the same faculty experts to repeat the pilot courses and recruited five faculty experts to teach the two additional sessions. We also provided lunch to maximize participation.

## Output

We considered attendance and session evaluations as outputs, early indicators that we were on-target to achieve our goals. This information also helped us identify areas for improvement.

### Attendance

An average of 30 fellows attended each session over 3 years. The fellows varied from session to session and year to year. A majority of the fellows were able to attend at least three out of the six sessions per year. Based on comments in the evaluations, informal feedback from fellows, and frequent requests for lecture materials when the session was missed, we determined that the greatest barrier to attendance was scheduling conflicts with other conferences and patient care.

We administered individual evaluations at the end of each session. On a scale of 1–5, fellows were asked to rate 1) the quality of the presentation 2) how well the objectives were achieved, and 3) the quality of the speaker. The average response rate for these session evaluations was 83%. The average scores over 3 years for the quality of the presentation, achievement of session objectives, and quality of the speaker were 4.70, 4.54, and 4.72, respectively.

We also used information from individual sessions to make two main improvements, both related to over-arching concerns about time. First, learning objectives were streamlined to fit more appropriately into a 1-hour time frame. Second, the curriculum was offered earlier in the academic year so that pertinent sessions were complete prior to the typical job application process of senior fellows.

## Outcome

We considered outcomes at the level of the fellow, the faculty, and at the level of the curriculum. We used perceived increases in knowledge, and how this knowledge was utilized as two indicators that we had met our goal of preparing fellows to transition to faculty. To determine outcomes, we used data from three time points: post-session evaluations described above, overall curriculum evaluations, and a 6-month follow-up survey. We also interviewed faculty members, who served as instructors for the course.

### Immediate session outcomes

We examined the item from session evaluations that asked fellows about their pre- and post-session knowledge regarding learning objectives. Paired *t*-test analysis revealed that for each objective, fellows reported feeling significantly more knowledgeable after the session, compared to before the session every year. Table 2 provides an example taken from an individual-session evaluation. Table 3 shows aggregated gains in knowledge per session per year.

**Table 2:** Example of perceived level of knowledge according to session objectives

Understanding different academic pathways				

Learning objectives	Before (Mean)	After (Mean)	*p*	*n*
Describe the roles, responsibilities, and academic expectations of a Clinician Educator and Clinician Researcher	2.5	4.2	0.0001	22
Identify strategies for achieving academic success as a Clinician Educator and Clinician Researcher	2.4	4.0	0.0001	23
Describe obstacles and challenges in each pathway	2.6	4.2	0.0001	24
Recognize meaningful achievement in each pathway	2.5	4.1	0.0001	23

**Table 3:** Aggregate gains in knowledge per session per year

	Perceived knowledge gained		
	
Topic	2011 cohort	2012 cohort	2013 cohort
Understanding academic pathways	32.6	22.2	25.6
Pitfalls of manuscript writing	30.5	20.4	23.5
Grant writing	23.0	28.6	28.4
Productivity and promotion	40.4	35.4	36.3
Interviewing and negotiating	26.7	28.4	22.5
Basics of Medical Employment Contracts	31.6	33.7	35.9

The perceived knowledge gained in each cell represents the difference in self-assessed level (%) of overall knowledge before and after each educational session. All results are statistically significant on paired *t*-test (*p*<0.05).

### Short-term course outcomes

We sent an on-line course evaluation to all participating fellows 1 week after the final session. Fellows were asked questions directly in line with the goal of the curriculum, whether the curriculum should be offered again, and to provide overall comments. Averaged over 3 years, course evaluations were favorable: 91% of respondents reported that the program helped them prepare for transitioning to an academic faculty position; and 94% agreed that the program should be offered to future fellows. We were heartened by comments such as, ‘Excellent! This has been much needed for a long time. Thank you for starting it’ and ‘There were several things that I didn't know and questions I didn't know who could answer. I learned about resources that would be extremely helpful that haven't been told to me. Definitely should continue to be offered’.

### Outcomes at 6-month follow-up

We sent an on-line 6-month follow-up survey to fellows who participated in the course. Anticipating that job applications would be complete for graduating fellows, we sent the survey in the spring and asked how fellows used information gained in the sessions to prepare for their own career transitions. Most respondents had attended at least three of the six sessions offered in the course.

Fellows expressed positive regard for the program in terms of its effectiveness in preparing them for transition to faculty. A majority of the fellows (58%) used the knowledge acquired in the curriculum to prepare for professional goals and advancement. Nearly all third-year fellows (94%) used this knowledge to apply for jobs. Fellows also used knowledge to inform decisions related to choice of scholarly projects and academic tracks and to improve their scholarly writing. The few fellows who had not applied acquired knowledge attributed this to not having a relevant opportunity.

### Impact on faculty

Appreciating the potential impact on faculty, we interviewed 6 core faculty members, who participate yearly in order to obtain their perspectives regarding reasons for success of the program, insights on their contributions to the program, motivating factors, and needed changes in the future. In sum, core faculty valued their participation because it brought personal reward and a sense of contributing to a larger purpose. As one said, ‘I am translating my skills into something larger. This one hour is magnified in a lot of ways and it's my way of helping people’. Faculty committed their time not only because they thought the curriculum was addressing a knowledge gap, but also because they identified themselves as teachers with relevant expertise. For example, faculty comments include, ‘The knowledge [I present] is critical to future careers; the essential nature of it makes it different from other courses’ and ‘I've contributed by opening their minds, and helping them to look at things [careers] broadly’.

## Discussion and lessons learned

As fellows across the globe complete their last stage of training and approach the final transition to faculty, they have a need to know about how to prepare for academic careers and are ready to learn. Transitioning to faculty is a shared experience across all countries, and our efforts to prepare our fellows can be replicated and built upon by educators internationally. Consistent with Knowles’ principles of adult learning (14), we provided instruction with high relevance and immediate applicability to real life experiences. In doing so, we were effective in enabling fellows to acquire new knowledge and to prepare for their new professional roles. Rather than seeking information on one's own through independent instruction or individual mentorship, an explicit curriculum such as ours can be developed to meet the adult learner's need to know.

Our curriculum has features similar to those of academic career programs in adult medicine and psychiatry (4–6, 8), indicating that the content of ACD programs may be transferable. The results from our experience support limited data from existing career development programs. Taken together, findings indicate that the development of dedicated and separate curricula is a useful intervention that enables fellows to be better equipped for academic careers. Similar to career development programs designed to attract medical students into academic pediatrics (15), this course may also influence fellows who are undecided about their future careers by previewing an academic career.

Using a logic model to frame our efforts, our report may serve as an evaluation roadmap for other programs, depicting how component parts logically flow toward the desired learning outcome. Based on robust evaluative methods, we consider this curriculum to be an effective and worthwhile investment of time and resources. Based on our experience, we offer these lessons learned:

1. The development and implementation of an ACD curriculum requires buy-in and continued collaboration among education leaders, program directors, faculty, and fellows.
2. Departments should designate time in the fellows’ already busy schedule for ACD curricula, time that may displace clinical or research responsibilities.
3. ACD curricula need dedicated faculty to serve as instructors and role models.

There are several limitations to our findings. Our evaluation did not have a comparison group and our response rate from fellows in the needs assessment was low. At present, we cannot link our educational intervention to careers. Furthermore, there may be variable exposures such as individual mentoring or division-specific instruction beyond the scope of this investigation. However, this report can inform educators on systematic curriculum development and evaluation in the arena of career transitioning and its positive impact on fellows and faculty.

## Conclusion

Using a logic model to organize our efforts, we systematically developed, implemented, evaluated, and revised an ACD curriculum for pediatric subspecialty fellows. Our curriculum enhances traditional fellowship education by using methods that translate well to adult learners, as they prepare for academic careers. Future directions include tracking the career paths and career satisfaction of the graduates of our pediatric fellowship programs.

## References

[1] Higgins R, Gallen D, Whiteman S (2005). Meeting the non-clinical education and training needs of new consultants. Postgrad Med J.

[2] Teunissen PW, Westerman M (2011). Opportunity or threat: the ambiguity of the consequences of transitions in medical education. Med Educ.

[3] Brown J, Ryland I, Shaw N, Graham D (2009). Working as a newly appointed consultant: as study into the transition from specialist registrar. Br J Hosp Med.

[4] Johnston B, Fenton C, Jain S (2002). A retreat for first-year medicine fellows. Acad Med.

[5] Levine SA, Caruso LB, Vanderschmidt H, Silliman RA, Barry PP (2005). Faculty development in geriatrics for clinician educators: a unique model for skills acquisition and academic achievement. J Am Geriatr Soc.

[6] McDonald FS, Schultz HJ, LaRusso NF (2002). A learner-centered academic career development curriculum. Acad Med.

[7] Medina-Walpole A, Fonzi J, Katz PR (2007). Academic career development in geriatric fellowship training. J Am Geriatr Soc.

[8] MacDonald J, Cole J (2004). Trainee to trained: helping senior psychiatric trainees make the transition to consultant. Med Educ.

[9] Department of Pediatrics, School of Medicine, Fellows College. http://pediatrics.ucsf.edu/fellows-college.

[10] Wilkie G, Raffaelli D (2005). In at the deep end: making the transition from SpR to consultant. Adv Psychiatr Treat.

[11] W.K. Kellogg Foundation (2004). Using logic models to bring together planning, evaluation, and action: logic model development guide.

[12] Balmer D, Schwartz A (2010). Innovation in pediatric residency education: the role of evaluation. Pediatrics.

[13] Campbell JR, Palazzi D, Rama JA, Hsu DC, Turner TL Building bridges between silos: how to build a program of intraprofessional training among pediatric fellows. Pediatr Acad Soc.

[14] Bennett, Elisabeth E, Blanchard, Rebecca D, Hinchey, Kevin T (2012). Applying Knowles’ andragogy to resident teaching. Acad Med.

[15] Smith WH, Rogers JG, Hansen TN, Smith CV (2009). Early career development in academic pediatrics of participants in the APS-SPR Medical Student Research Program. Pediatr Res.

